# Comprehensive genomic analysis of molecular residual disease based on circulating tumour DNA in postoperative patients with colorectal cancer

**DOI:** 10.1002/ctm2.70041

**Published:** 2024-10-18

**Authors:** Qingqi Hong, Jingtao Zhu, Hexin Lin, Yinan Chen, Haoyu Bai, Linghua Yan, Li Xiao, Jun You

**Affiliations:** ^1^ Department of Gastrointestinal Oncology Surgery The First Affiliated Hospital of Xiamen University School of Medicine Xiamen University Xiamen China; ^2^ The School of Clinical Medicine Fujian Medical University Fuzhou China; ^3^ Department of Innovative Technology Shanghai Tongshu Biotechnology Research Institute Shanghai China; ^4^ Department of Oncology Zhongshan Hospital of Xiamen University School of Medicine Xiamen University Xiamen China

Dear Editor,

Colorectal cancer (CRC) is a major global health issue with a 40% 5‐year mortality rate,^1^ and 30%−50% of patients still experience recurrence after curative resection.^2^, ^3^ The clinical utility of molecular residual disease (MRD) detection in risk stratification and recurrence detection using circulating tumour DNA (ctDNA) noninvasively has attracted widespread attention but with limited studies.^4^, ^5^, ^6^, ^7^ We measured the value of tumour‐agnostic ctDNA‐guided MRD for recurrence prediction and monitoring, and explored the biological characteristics of ctDNA from MRD.

We included 104 patients with stage I‐IV CRC (Figure S1), aged 26 to 82 years. Fourteen (13.5%) patients had positive landmark MRD, and thirty‐two (30.8%) patients had positive longitudinal MRD (Table S1). The most frequently variant genes in tumour tissue were *TP53*, *KRAS* and *APC* (Figure S2A), while in ctDNA were *KRAS*, *EGFR* and *TP53* (Figure S2B).

Patients with negative landmark MRD had a lower recurrence percentage than positive cases (11.1% vs. 57.1%, *p* < .001), yielding a negative predictive value (NPV) of 88.9% (Figure 1A). The latter group had a significantly higher recurrence risk (hazard ratio [HR], 7.325; *p* < .001, Figure 1B). As for longitudinal MRD, the recurrence percentage was higher in positive‐MRD compared to negative‐MRD (43.8% vs. 5.6%, *p* < .001), yielding a high NPV (94.4%, Figure 1C). Positive longitudinal MRD was correlated with an elevated risk of recurrence (HR: 9.385; *p* < .001, Figure 1D). In multivariate analysis for disease‐free survival (DFS), landmark and longitudinal MRD‐positive were significantly associated with increased recurrence risks (Figure 1E). Furthermore, we explored the effect of longitudinal MRD status in patients receiving and not receiving neoadjuvant therapy (NAT). In both groups, longitudinal positive‐MRD patients had higher recurrence risk than negative‐MRD patients (NAT: HR, 12.509, *p* = .011; non‐NAT: HR, 9.611, *p* < .001; Figure S3A and B). The recurrence risk was lower in those with negative landmark ctDNA and negative longitudinal ctDNA (Figure S4A–D). Although the NPV for ctDNA was slightly higher than that for MRD, the PPV for ctDNA was lower than that for MRD. The area under the curve (AUC) of 3‐year DFS for landmark MRD was  .678, longitudinal MRD was  .895, and landmark and longitudinal MRD had higher AUCs than ctDNA (Figure S4E). Fourteen out of 18 cases with recurrence detected MRD at the postoperative time points (Figure 1F). And 85.7% (12/14) patients had positive MRD detection before computed tomography confirmed recurrence (median: 198.5 days, Figure 1G). For MRD‐positive cases, actions such as initiating adjuvant therapies, more aggressive treatment, intensified monitoring, or early intervention before clinical relapse occurs, may be clinically undertaken, potentially leading to better disease control, improved survival rates, and enhanced quality of life.

**FIGURE 1:**
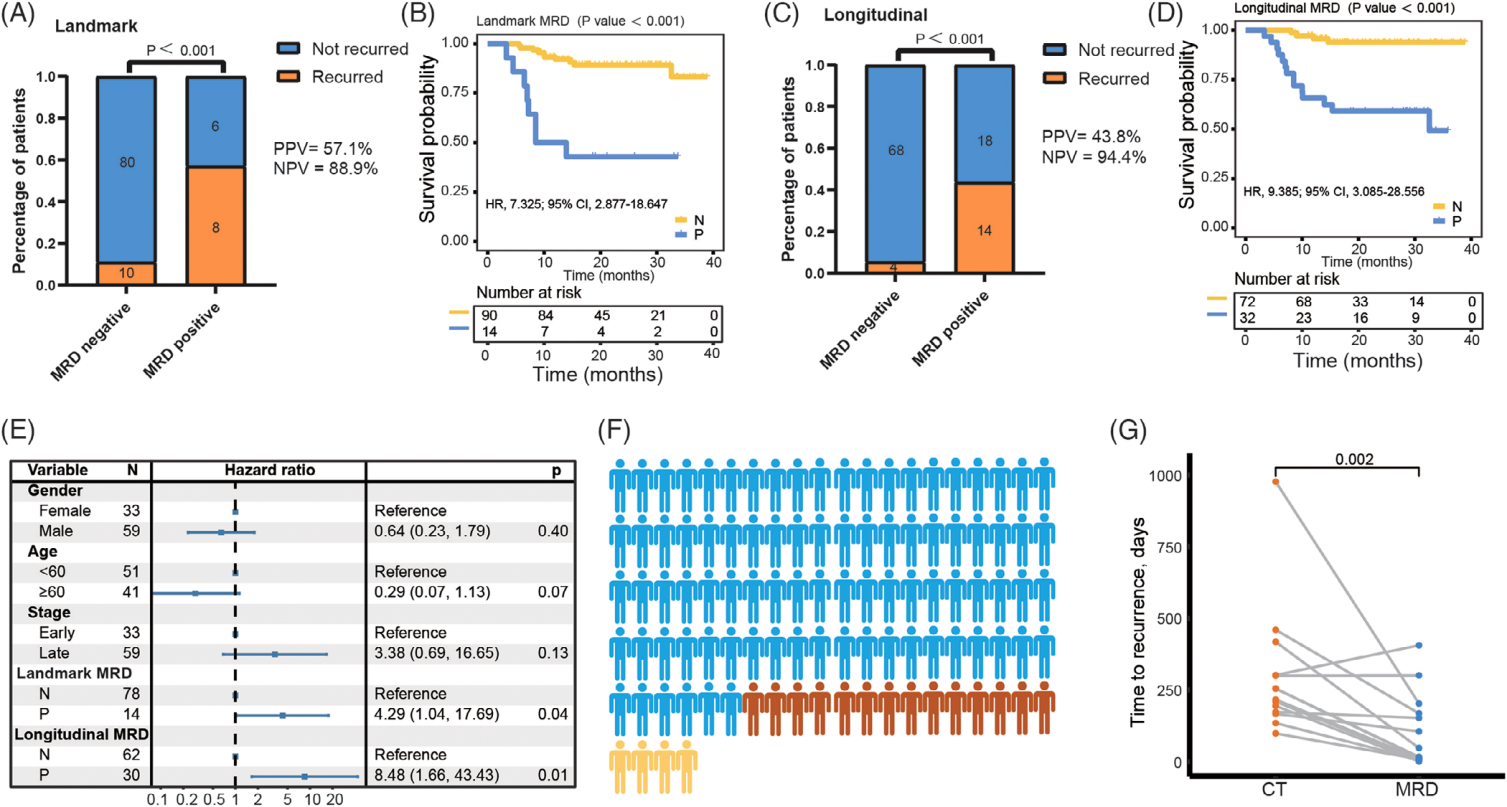
Prognostic significance of MRD in patients with CRC. (A) Bar graph illustrates recurrence percentages according to MRD status at landmark time point (chi‐square test). Patients with negative MRD had a lower recurrence percentage than positive cases. The postoperative landmark MRD status was determined from the first ctDNA sequencing of a blood sample taken within 2 months after surgical resection. (B) Kaplan–Meier survival analysis of DFS stratified by MRD‐negative and MRD‐positive at landmark time point (log‐rank test). (C) Bar graph illustrates recurrence percentages according to MRD status at longitudinal time points. The recurrence percentage was higher in positive‐MRD compared to negative‐MRD. Longitudinal MRD positivity was defined as the positive MRD detected at any postoperative time point, while longitudinal MRD negativity referred to having negative MRD at all time points (chi‐square test). (D) Kaplan–Meier survival analysis of DFS stratified by MRD‐negative and MRD‐positive at longitudinal time points (log‐rank test). (E) Forest plot depicting the multivariate analysis (including gender, age, stage, landmark MRD status, and longitudinal MRD status) for DFS in patients with CRC (Cox Proportional Hazards Model). MRD‐positive landmark and longitudinal time points showed significant association with increased recurrence risks. (F) Diagram illustrating the number of patients with recurrence and positive MRD detection. Yellow represents individuals with recurrence but no positive MRD detection. Orange represents individuals with recurrence and positive MRD detection. (G) Comparison of time to relapse by MRD positivity and standard‐of‐care CT (Wilcoxon test). CT, computed tomography; NPV, negative predictive value; N, negative; P, positive; PPV, positive predictive value.

Genetic markers showed complex interactions with clinical outcomes. *KRAS*, *APC*, *FGFR2* and *RET* variants in ctDNA were associated with inferior DFS (Figure 2A–D). In tissue, *KRAS* and *PIK3CA* variants may have the prediction potential for DFS (both *p* > .05, Figure 2E and F). The recurrence percentage in patients with *KRAS* variants in cell‐free DNA (cfDNA_*KRAS+*) was significantly higher than those without (Figure 2G). Patients at an earlier stage exhibited a significantly better DFS (Figure S5). In the negative landmark MRD group, patients with metastases had higher variant frequencies of *KRAS*, *ALK*, *RET* and *NTRK2* than those without (Table S2). A similar trend for these genes was observed in longitudinal MRD‐negative patients (Table S3). The tumour mutation burden did not exhibit predictive capability for DFS, while the patients with high microsatellite instability tended to have a better DFS (Figure 2H and I).

**FIGURE 2:**
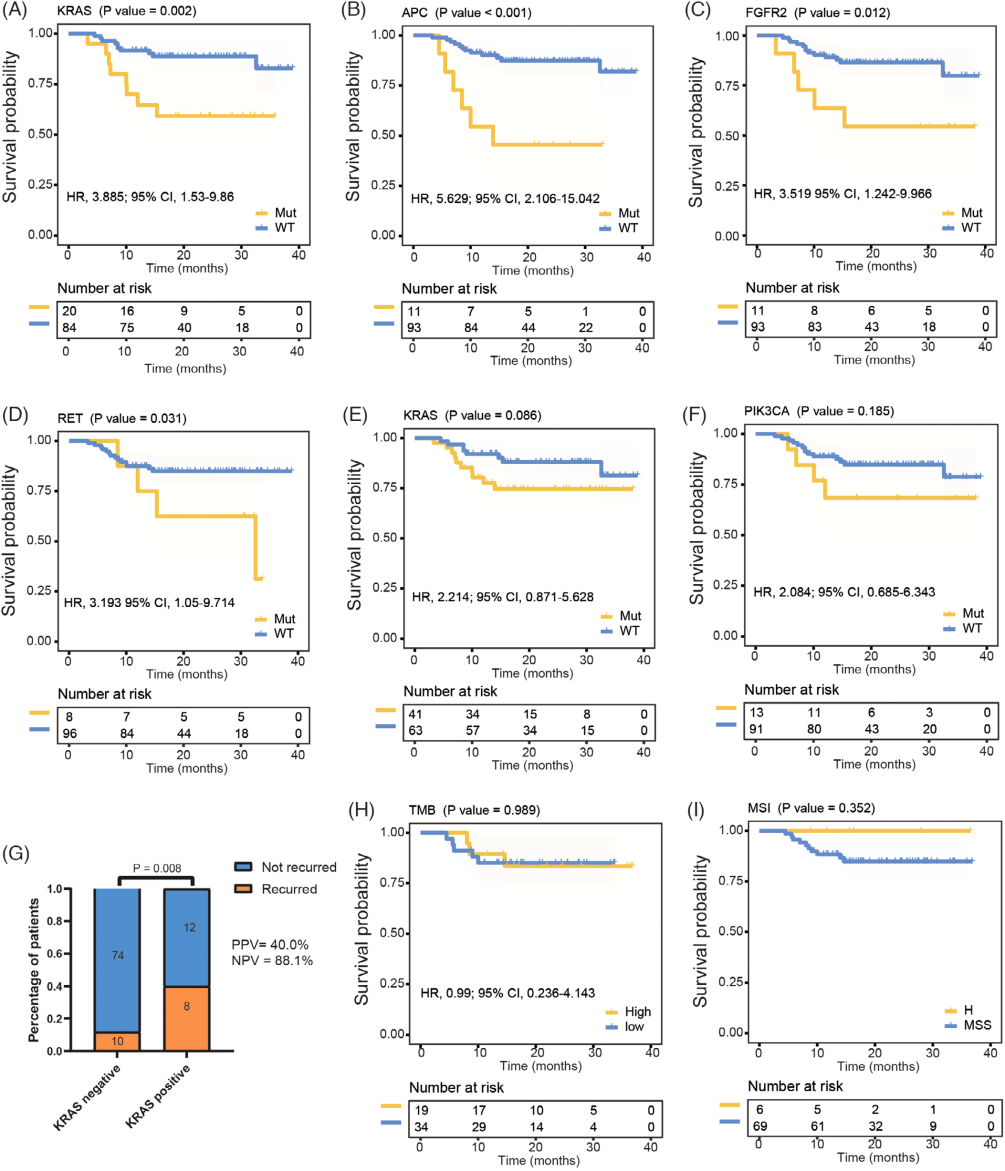
Association of genetic characteristics with DFS. (A–D) Kaplan–Meier survival analysis of DFS stratified by *KRAS*, *APC*, *FGFR2*, and *RET* variants in cfDNA (log‐rank test). (E, F) Kaplan–Meier survival analysis of DFS stratified by *KRAS* and *PIK3CA* variants in tumour tissue samples (log‐rank test). (G) Bar graph displays recurrence percentages by cfDNA_*KRAS* variants (cfDNA_*KRAS*+, chi‐square test). (H) Kaplan–Meier survival analysis of DFS stratified by TMB‐high and TMB‐low (log‐rank test). (I) Kaplan–Meier survival analysis of DFS stratified by MSS and MSI‐H (log‐rank test). MSS, microsatellite stability; MSI‐H, high microsatellite instability; TMB, tumour mutational burden.

The combination of MRD status with cfDNA_*KRAS+* was significantly associated with patients’ outcomes. Among patients with positive landmark MRD and positive longitudinal MRD, cfDNA_*KRAS+* showed a trend to poorer DFS (Figure 3A and B). In patients with negative landmark MRD, cfDNA_*KRAS+* was associated with poorer DFS (Figure 3A). Then, patients with a positive result in at least one of the 2 statuses (cfDNA_*KRAS* variants and MRD) were designated as positive. The combination of landmark MRD and cfDNA_*KRAS+*, and the combination of longitudinal MRD and cfDNA_*KRAS+* were significantly associated with inferior DFS (Figure 3C and D). For the 3‐year DFS, the combination of landmark MRD and cfDNA_*KRAS+* reached an AUC of  .768, surpassing both landmark MRD (.678) and cfDNA_*KRAS+* (.681). Similarly, the integration of longitudinal MRD and cfDNA_*KRAS+* reached an AUC of  .922, surpassing both longitudinal MRD (.895) and cfDNA_*KRAS+* (.681) (Figure 3E).

**FIGURE 3:**
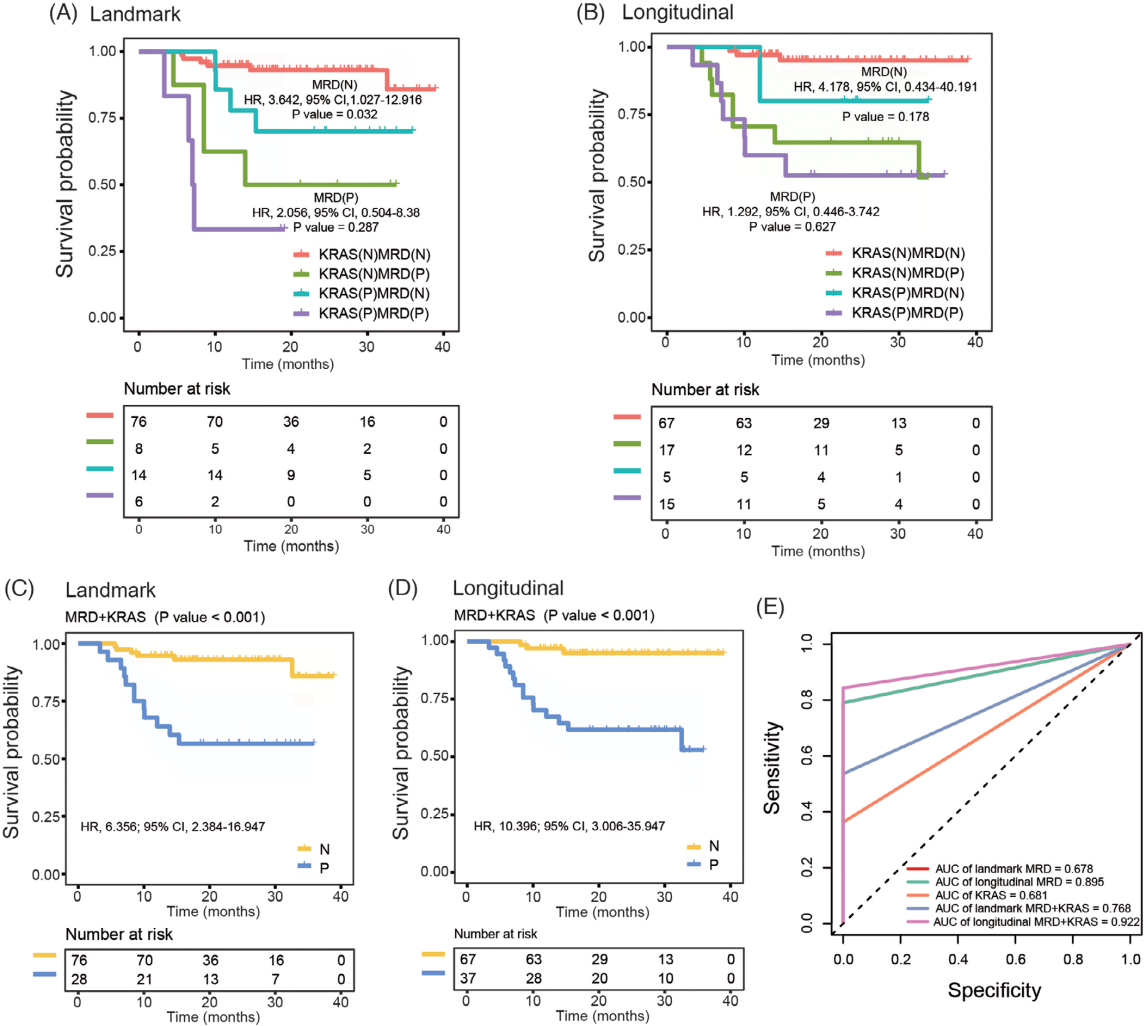
Association of the combination cfDNA_*KRAS* variants and MRD status with DFS. (A) Kaplan–Meier analysis comparing DFS between landmark MRD‐positive patients with and without cfDNA_*KRAS* variants, and comparing DFS between landmark MRD‐negative patients with and without cfDNA_*KRAS* variants (log‐rank test). (B) Kaplan–Meier analysis comparing DFS between longitudinal MRD‐positive patients with and without cfDNA_*KRAS* variants, and comparing DFS between longitudinal MRD‐negative patients with and without cfDNA_*KRAS* variants (log‐rank test). (C, D) Kaplan–Meier survival analysis of DFS stratified by the combination of cfDNA_*KRAS* variants and landmark MRD status, and the combination of cfDNA_*KRAS* variants and longitudinal MRD status (log‐rank test). MRD+*KRAS* negative, both MRD status and *KRAS* variants were negative; MRD+*KRAS* positive, either MRD status or *KRAS* variants tests showed positivity. (E) Receiver operator characteristic (ROC) curve analysis for 3‐year DFS based on landmark MRD status, longitudinal MRD status, cfDNA_*KRAS* variants, the combination of cfDNA_*KRAS* variants and landmark MRD status, and the combination of cfDNA_*KRAS* variants and longitudinal MRD status. The combination of MRD and cfDNA_*KRAS* variants achieved higher AUCs for 3‐year DFS, outperforming individual tests. N, negative; P, positive; AUC, area under the curve.

The recurrence risk of MRD‐positive and MRD‐negative patients stratified by the adjuvant therapy (AT) was analysed. No survival benefit was observed from AT in landmark MRD‐positive patients due to the limited number of patients without AT (*n* = 2; Figure S6A and C). Patients with AT tended to have shorter DFS and higher percentage of recurrence than patients without AT in the landmark MRD‐negative group (Figure S6B and D). Adjuvant therapy may not be suitable for landmark MRD‐negative CRC patients to prevent over‐medicalisation.

The I/II class mutation sites and all mutation sites were predominantly concentrated in fragments below 200 bp (Figure 4A and E). Furthermore, we compared the proportion of mutation reads at different length intervals. The proportions of class I/II mutation reads and all mutation reads in the fragments ≤ 100 bp were significantly higher than those in the fragments >100 bp (Figure 4B and F). The percentages of fragments ≤ 100 bp in class I/II mutation reads and all mutation reads were higher than those in non‐mutated reads (Figure 4C and G). The median length of class I/II mutation reads was lower than non‐mutated reads (163 bp vs. 168 bp; Figure 4D). The median length of all mutation reads was also lower (166 bp vs. 169 bp; Figure 4H). Mutation sites were enriched in shorter fragments, which could potentially improve cfDNA detection methods, guiding the risk stratification, prognostic prediction, and monitoring of disease recurrence.

**FIGURE 4:**
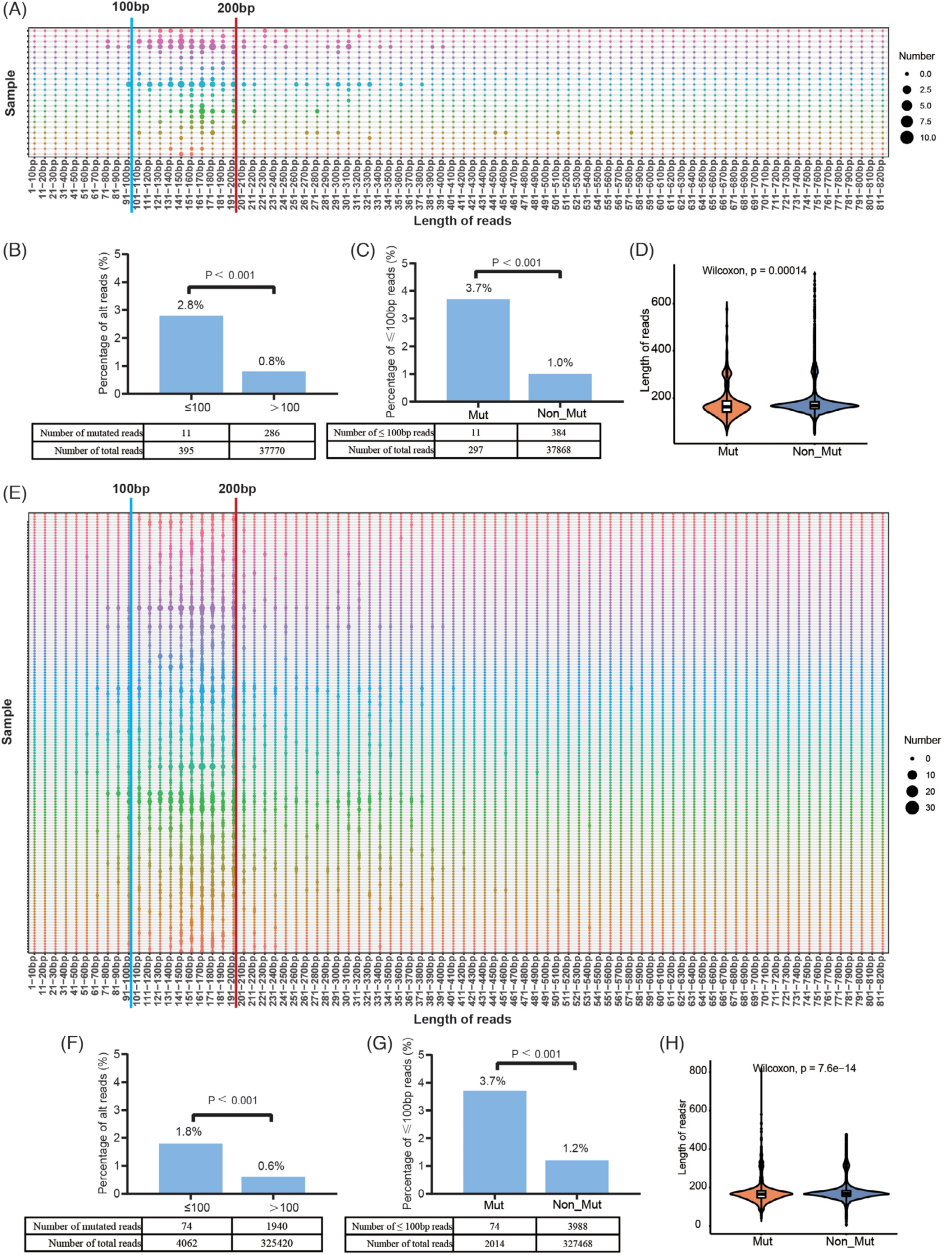
The characteristic of mutation reads in cfDNA. (A) The distribution of class I/II mutation reads in cfDNA at different lengths. Reads predominantly concentrated in fragments below 200 bp. (B) A comparison of the proportion of class I/II mutation reads at different intervals (chi‐square test). The proportions of mutation reads in the fragments ≤ 100 bp were significantly higher than those in the fragments >100 bp. (C) A comparison of the proportion of reads ≤100 bp in class I/II mutation reads and non‐mutated reads (chi‐square test). The percentages of fragments ≤ 100 bp in mutation reads were higher than those in non‐mutated reads. (D) A comparison of the length of reads in class I/II mutation reads and non‐mutated reads (Wilcoxon test). The median length of class I/II mutation reads was lower than non‐mutated reads (163 bp vs. 168 bp). (E) The distribution of all mutation reads in cfDNA at different lengths. Reads predominantly concentrated in fragments below 200 bp. (F) A comparison of the proportion of all mutation reads at different intervals (chi‐square test). The proportions of mutation reads in the fragments ≤ 100 bp were significantly higher than those in the fragments >100 bp. (G) A comparison of the proportion of reads ≤100 bp in all mutation reads and non‐mutated reads (chi‐square test). The percentages of fragments ≤ 100 bp in mutation reads were higher than those in non‐mutated reads. (H) A comparison of the length of reads in all mutation reads and non‐mutated reads (Wilcoxon test). The blue line represents read length at 100 bp, and the red line represents read length at 200 bp. The median length of all mutation reads was lower than non‐mutated reads (166 bp vs. 169 bp). Mut, mutated reads; Non_Mut, non‐mutated reads.

Our research underscored the remarkable prognostic value of ctDNA‐based MRD monitoring in postoperative CRC patients. Notably, integrating cfDNA_KRAS variants with MRD significantly enhanced the precision of risk stratification. Furthermore, our findings revealed a trend towards enriching mutation sites within shorter cfDNA fragments, offering pivotal insights for developing advanced cfDNA detection strategies.

## AUTHORS' CONTRIBUTIONS

Jun You, Li Xiao and Linghua Yan conceived of the study plan. Qingqi Hong, Jingtao Zhu, Hexin Lin, Yinan Chen, Haoyu Bai and Linghua Yan collected, analysed and interpreted data. Qingqi Hong, Jingtao Zhu, Hexin Lin, Yinan Chen and Haoyu Bai drafted the manuscript. Jun You, Li Xiao and Linghua Yan revised the manuscript. All authors reviewed the manuscript and had final approval of the submitted and published version.

## CONFLICT OF INTEREST STATEMENT

The authors have no conflict of interest to declare.

## ETHICS STATEMENT

This study was approved by the ethics committee of the corresponding hospital, and was carried out in compliance with the Declaration of Helsinki Principles. Written informed consent was obtained from all study participants.

## Supporting information



Supporting Information

## Data Availability

The datasets analysed during the current study are available from the corresponding author on reasonable request.
