# Supplementary material for: Comprehensive genomic analysis of molecular residual disease based on circulating tumour DNA in postoperative patients with colorectal cancer
Source: Clin Transl Med. 2024 Oct 18;14(10):e70041. doi: 10.1002/ctm2.70041 (PMC11488335; doi:10.1002/ctm2.70041)
Supplement: Supplementary file 1 — Supporting Information [file CTM2-14-e70041-s001.docx]

**Supplementary Materials**

**List of Supplementary Information**

**Materials and Methods**

**Supplementary Figures**

**Figure S1.** Flow diagram illustrating patient enrollment, sample collection, and subgroups for addressing the clinical questions. AT: adjuvant therapy, MRD: molecular residual disease, CT: computed tomography.

**Figure S2.** Genetic variant landscape of all enrolled patients.

**Figure S3.** Prognostic significance of longitudinal MRD in patients receiving neoadjuvant therapy and patients not receiving neoadjuvant therapy.

**Figure S4.** Prognostic significance of ctDNA in patients with CRC.

**Figure S5.** Association of clinicopathological factors with DFS.

**Figure S6.** Association of adjuvant therapy with DFS at distinct MRD status in all enrolled patients with CRC.

**Supplementary Tables**

**Table S1.** Relationship between clinicopathologic factors and MRD status.

**Table S2.** The comparison of gene variation frequency in cfDNA of the recurrent and non-recurrent groups from landmark MRD negative patients.

**Table S3.** The comparison of gene variation frequency in cfDNA of the recurrent and non-recurrent groups from longitudinal MRD negative patients.

**Materials and methods**

**Patients**

A total of 104 eligible patients with stage Ⅰ-Ⅳ CRC were enrolled in our study. The age of patients in this study ranged from 26 to 82 years old. All patients received surgical resection. Tumor tissue was collected at surgery, serial blood samples were collected postoperatively. The first blood sample was collected within 2 months after surgery surgical resection in each patient, another 1-2 postsurgical blood samples were collected in most patients. The ctDNA-based MRD testing was performed in each blood sample. The patient enrollment and subgroups for addressing the clinical questions are illustrated in the study flow chart (Figure S1). Disease-free survival (DFS) is defined as the time from surgical resection to recurrence of tumor or death. This study was approved by the ethics committee of the corresponding hospital and was performed in accordance with the Declaration of Helsinki.

**Sample preparation and ZhenXinan MRD NGS Panel sequencing**

Plasma and tissue samples were used for tumor DNA detection and leucocyte samples for germline DNA detection. The tumor DNA and matched leukocyte DNA were examined by a multi-gene sequencing panel. Tumor tissue DNA was extracted from formalin-fixed paraffin-embedded (FFPE) tissue samples using the tissue kit according to the manufacturer’s instructions (QIAGEN, Venlo, the Netherlands), and quantiﬁed by Qubit 4.0 using the dsDNA HS Assay Kit (ThermoFisher Scientiﬁc). Cell-free (cfDNA) was extracted from plasma using cell free nucleic acid extraction kit according to the manufacturers’ instructions (Tongshu Biotechnology Co., Ltd., Shanghai, China). A universal DNA library preparation kit (Tongshu Biotechnology Co., Ltd., Shanghai, China) was used to prepare libraries for the Illumina sequencing platform. The kit is double-stranded developed for Illumina's high-throughput sequencing platform based on the A-T linkage principle DNA library construction kit. The kit is compatible with liquid phase hybridization targeted capture sequencing. The kit is paired with Unique Dual Indexes (UDI) connectors to improve label hopping and label mismatch on the Illumina sequencing platform question. The kit contains magnetic beads for DNA purification and ADAPTS optimized fragment screening protocols. All reagents in this kit pass quality control and function validation to ensure the library's stable output. Library construction sample requirements: sample quality: gDNA, no serious degradation by agarose gel electrophoresis; FFPE DNA, fragment length > 500 bp; cfDNA, no large fragment contamination. Total sample size: 1 ~ 500 ng; Qubit or quantitative PCR is recommended for quantification. Sample purity: OD260/OD280 = 1.8 ~ 2.0; OD260/OD230 = 2.0 to 2.5. ZhenXinan MRD NGS Panel (Tongshu Biotechnology Co., Ltd., Shanghai, China) adequately covering oncodriver genes was used to identify mutation. The ctDNA recovery efficiency of the Tongshu extraction kit is higher, which makes the input amount of ctDNA higher. The ctDNA extraction kit can obviously remove long fragments (gDNA pollution), and retain more short fragments of ctDNA. The advantages of the above two aspects make ctDNA can effectively filter background noise during the sequencing process, significantly increase the effective sequencing depth of ctDNA, and increase the detection rate. Sequencing was performed as paired-end (PE) reads on the Illumina Novaseq 6000 platform using PE150 sequencing chemistry, with mean sequencing depths of more than 10000×. The detection limit of DNA mutation detection is 0.1%

**Data processing**

The sequence data were filtered by Fastp and aligned to the human reference genome (hg19) using Burrows-Wheeler Aligner (BWA, Version: 0.7.12-r1039). Single nucleotide variants (SNVs) and insertion/deletion (Indel) were detected using vardict-java and Mutect2 of GATK4 (version4.1.8.1). Structural variations were detected using factera. After filtering, those variants were then annotated by ensembl’s Variant Effect Predictor (VEP, version 104). Gene mutations were identified and annotated using self-built bioinformatics analysis methods. Somatic variants were classified into four tiers based on their level of clinical significance, as reported previously[1]. MRD positive was defined as the detection of at least one class I/II driver gene variant in cfDNA (tier I, variants with strong clinical significance; tier II, variants with potential clinical significance), which are commonly associated with tumorigenesis. ctDNA positive was defined as the detection of at least one gene variant in cfDNA. The postoperative landmark MRD status was determined from the first cfDNA sequencing of a blood sample taken within 2 months after surgical resection. The postsurgical longitudinal MRD positivity was defined as the positive MRD detected at any postoperative time point, while longitudinal MRD negativity referred to having negative MRD at all time points.

**Statistical analysis**

Fisher's exact test or chi-square test was employed to compare the frequency data between two groups. The Kaplan-Meier survival analysis was performed, and the resulting curves were compared by the log-rank test. HRs and 95% CIs were calculated using the univariate Cox analysis. For all statistical tests, P < 0.05 was considered statistically significant.


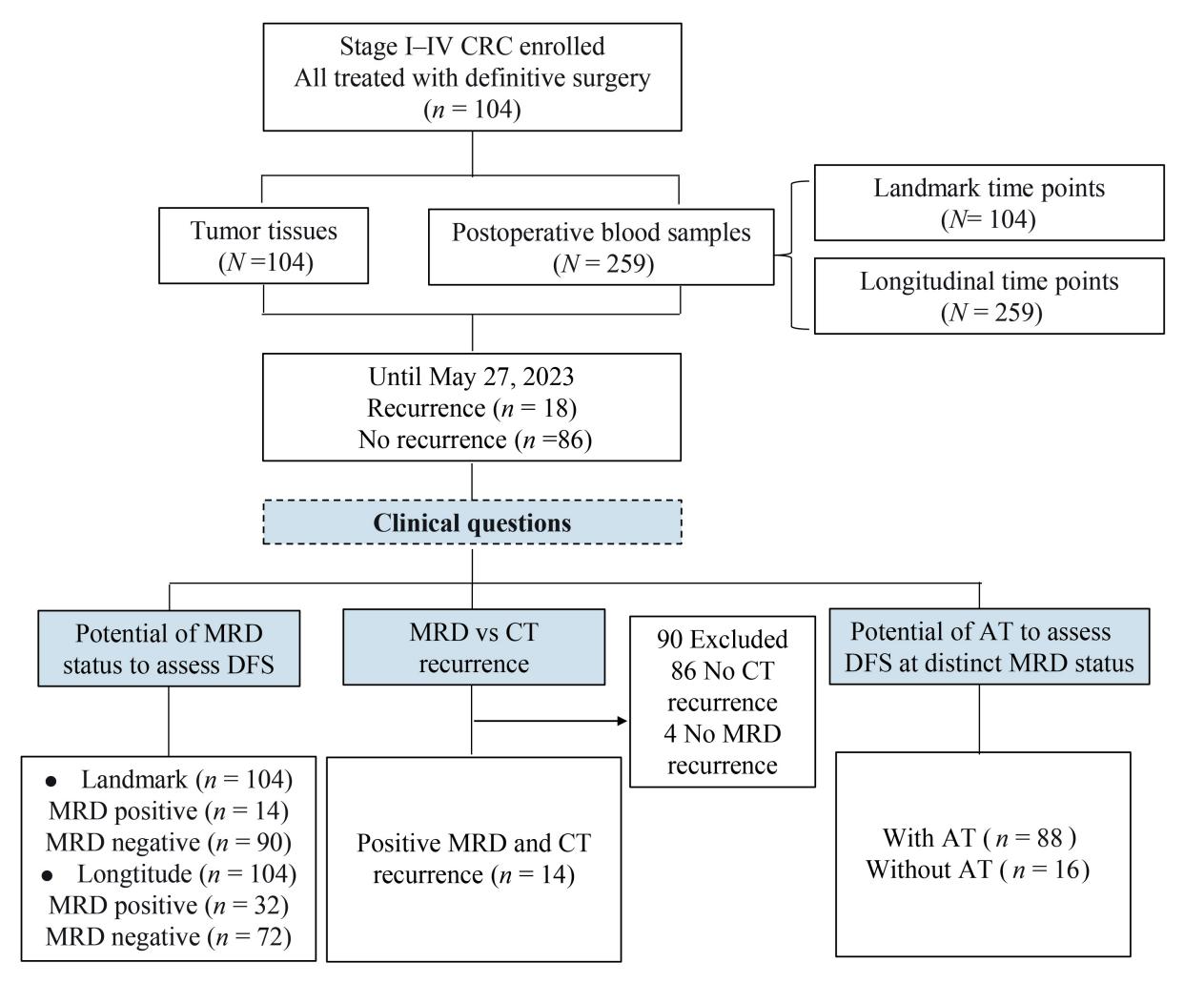


**Figure S1.** Flow diagram illustrating patient enrollment, sample collection, and subgroups for addressing the clinical questions. The postoperative landmark MRD status was determined from the first ctDNA sequencing of a blood sample taken within 2 months after surgical resection. Longitudinal MRD positivity was defined as the positive MRD detected at any postoperative time point, while longitudinal MRD negativity referred to having negative MRD at all time points. AT, adjuvant therapy; MRD, molecular residual disease; CT, computed tomography.


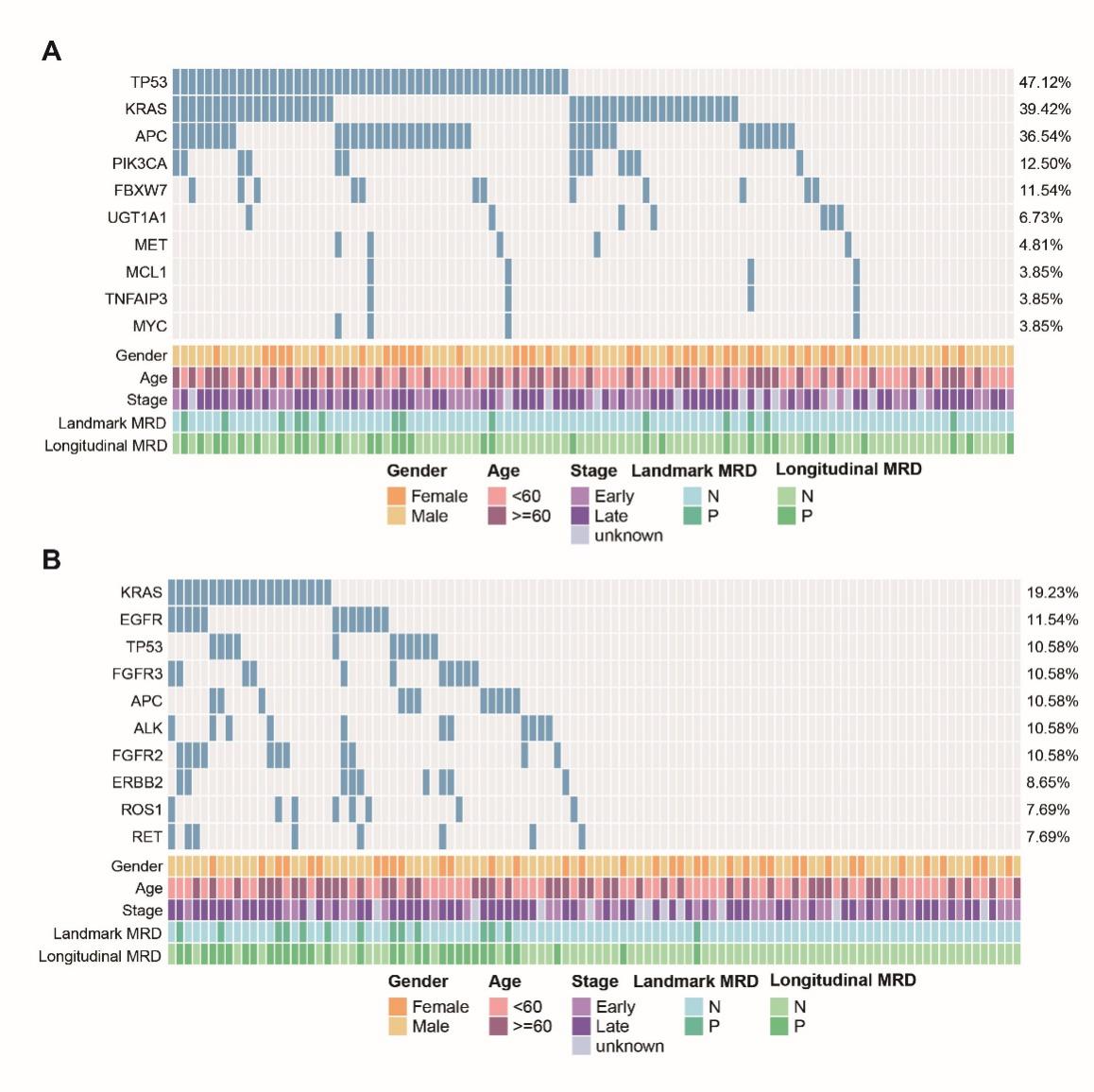


**Figure S2.** Genetic variant landscape of all enrolled patients. (A) The top 10 gene variant landscape of tumor tissue samples. The most frequent variant genes were *TP53*, *KRAS*, and *APC*. (B) The top 10 gene variant landscape of blood samples. The most commonly mutated genes were *KRAS*, *EGFR*, and *TP53*. N, negative; P, positive.


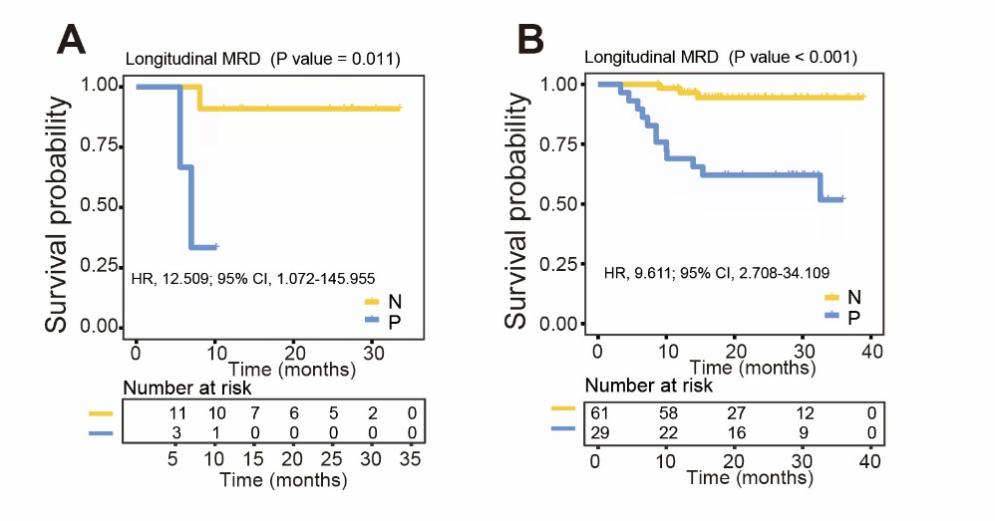


**Figure S3.** Prognostic significance of longitudinal MRD in patients receiving neoadjuvant therapy and patients not receiving neoadjuvant therapy. (A) Kaplan-Meier survival analysis of DFS stratified by MRD-negative and MRD-positive at longitudinal time points in patients receiving neoadjuvant therapy. (B) Kaplan-Meier survival analysis of DFS stratified by MRD-negative and MRD-positive at longitudinal time points in patients not receiving neoadjuvant therapy. MRD, molecular residual disease; N, negative; P, positive.


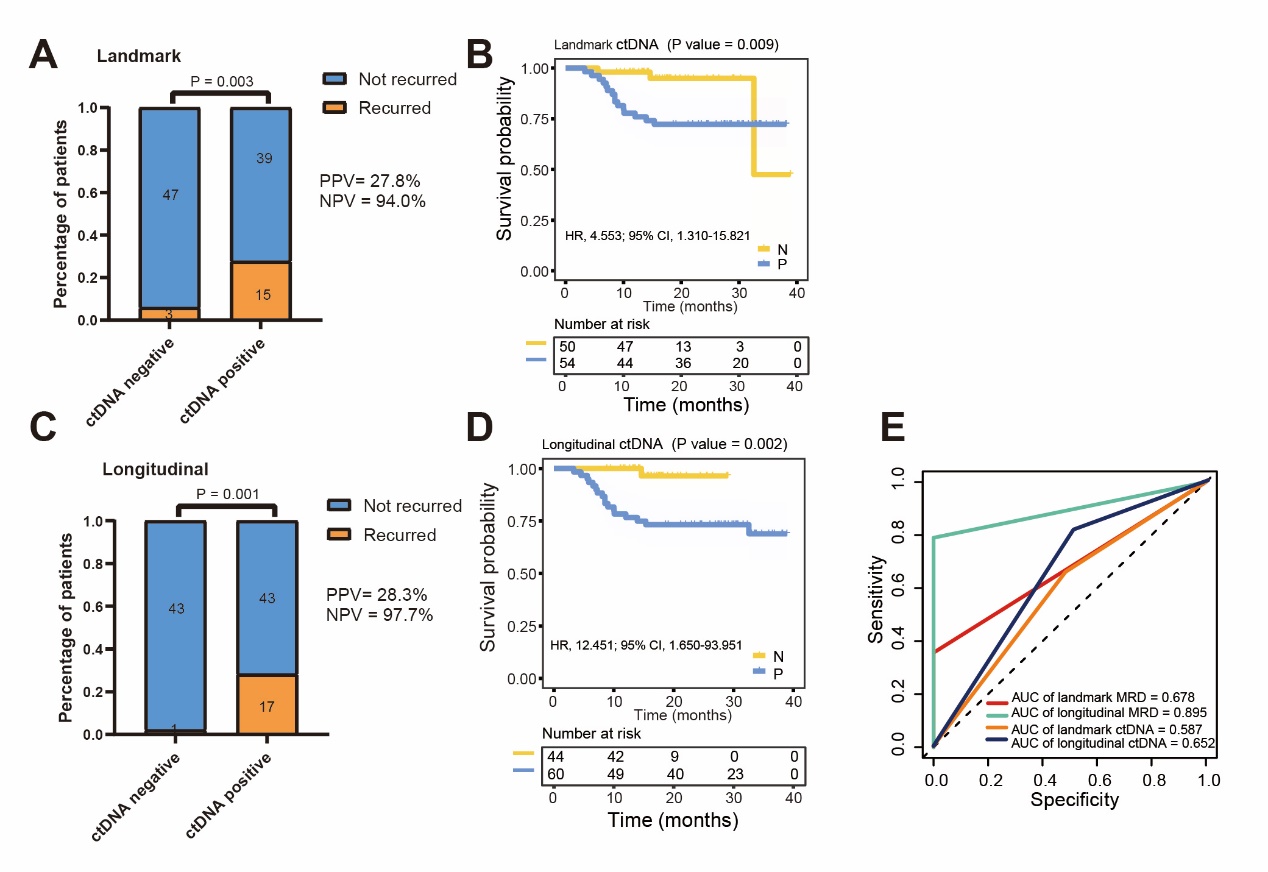


**Figure S4.** Prognostic significance of ctDNA in patients with CRC. (A) Bar graph illustrates recurrence percentages according to ctDNA status at landmark time point (chi-square test). Patients with negative ctDNA had a lower recurrence percentage than positive cases. (B) Kaplan-Meier survival analysis of DFS stratified by ctDNA-negative and ctDNA-positive at landmark time point. (C) Bar graph illustrates recurrence percentages according to ctDNA status at longitudinal time points (chi-square test). Patients with negative ctDNA had a lower recurrence percentage than positive cases. (D) Kaplan-Meier survival analysis of DFS stratified by ctDNA-negative and ctDNA-positive at longitudinal time points. (E) ROC analysis for 3-year DFS based on landmark MRD status, longitudinal MRD status, landmark ctDNA status, and longitudinal ctDNA status. Longitudinal MRD status achieved a higher AUC for 3-year DFS than other tests. N, negative; P, positive; NPV, negative predictive value; PPV, positive predictive value; AUC, the area under the curve.


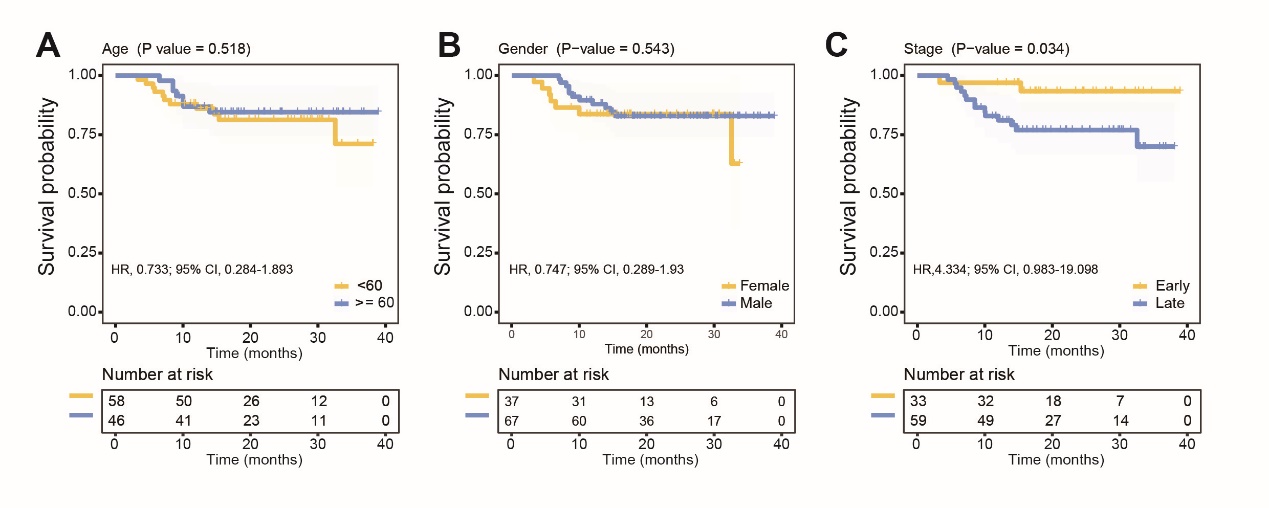


**Figure S5.** Association of clinicopathological factors with DFS. (A-C) Kaplan-Meier survival analysis of DFS stratified by age (<60 or ≥60), gender, and stage (early, I/II; late, III/IV) (log-rank test).


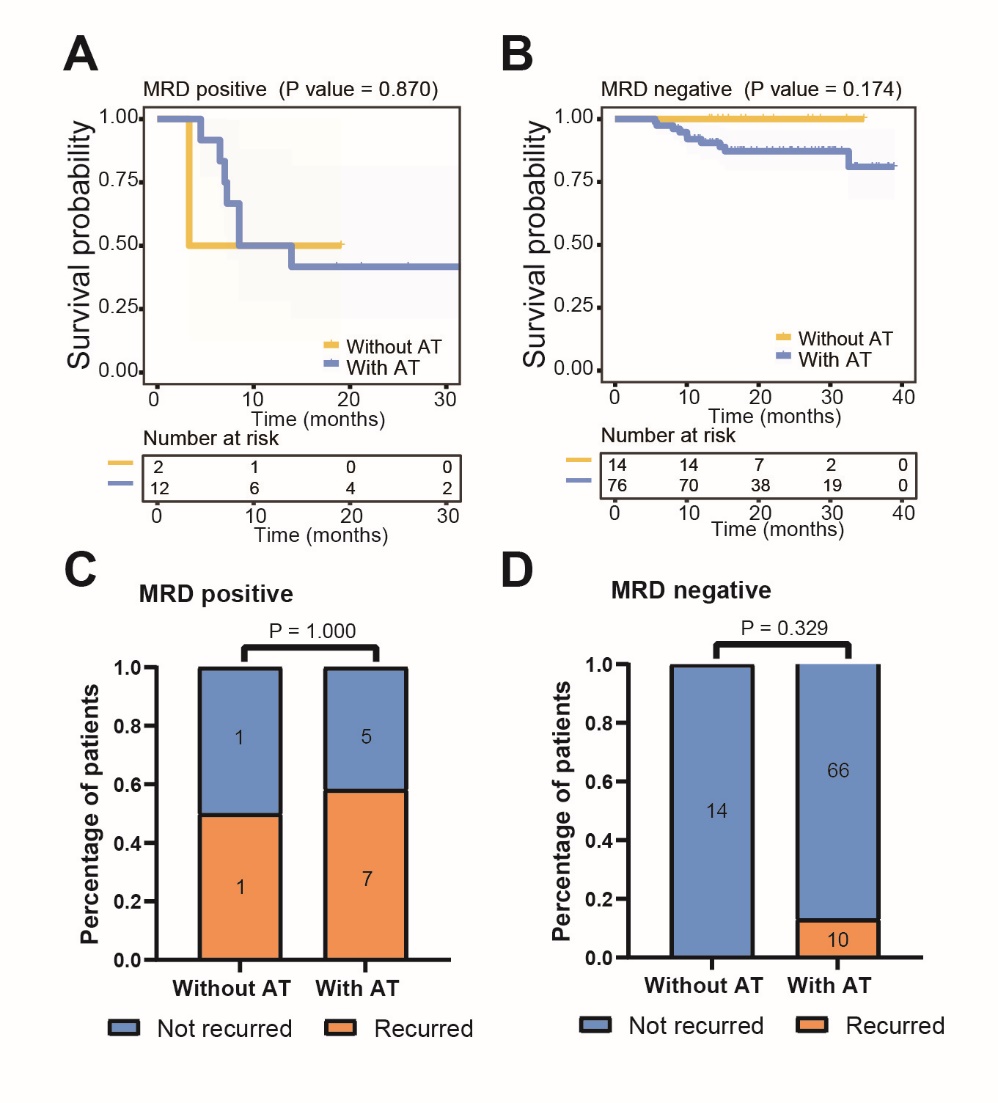


**Figure S6.** Association of adjuvant therapy with DFS at distinct MRD status in all enrolled patients with CRC. (A-B) Kaplan–Meier survival analysis of DFS stratified by adjuvant therapy in patients with positive landmark MRD (A), and patients with negative landmark MRD (B) (log-rank test). (C-D) Bar graph displays recurrence percentages by adjuvant therapy in patients with positive landmark MRD, and patients with negative landmark MRD (chi-square test). AT: adjuvant therapy.

**Table S1**. Relationship between clinicopathologic factors and MRD status.

| **Variables** | **Landmark MRD negative (n=90)** | **Landmark MRD positive (n=14)** | **Fisher’s exact test**  **(*P*)** | **Longitudinal MRD negative (n=72)** | | **Longitudinal MRD positive (n=32)** | **Fisher’s exact test**  **(*P*)** |
| --- | --- | --- | --- | --- | --- | --- | --- |
| **Gender** |  |  |  |  |  | |  |
| Female | 31 (34.4%) | 6 (42.9%) | 0.56 | 25 (34.7%) | 12 (37.5%) | | 0.826 |
| Male | 59 (65.6%) | 8 (57.1%) |  | 47 (65.3%) | 20 (62.5%) | |  |
| **Age** |  |  |  |  |  | |  |
| <60 | 53 (58.9%) | 5 (35.7%) | 0.148 | 40 (55.6%) | 18 (56.3%) | | 1 |
| ≥60 | 37 (41.1%) | 9 (64.3%) |  | 32 (44.4%) | 14 (43.8%) | |  |
| **Stage** |  |  |  |  |  | |  |
| Ⅰ | 4 (4.4%) | 0 (0.0%) | 0.444 | 3 (4.2%) | 1 (3.1%) | | 0.355 |
| Ⅱ | 26 (28.9%) | 3 (21.4%) |  | 23 (31.9%) | 6 (18.8%) | |  |
| Ⅲ | 41 (45.6%) | 8 (57.1%) |  | 30 (41.7%) | 19 (59.4%) | |  |
| Ⅳ | 7 (7.8%) | 3 (21.4%) |  | 6 (8.3%) | 4 (12.5%) | |  |
| **MSI** |  |  |  |  |  | |  |
| H | 6 (6.7%) | 0 (0.0%) | 1 | 5 (6.9%) | 1 (3.1%) | | 1 |
| MSS | 60 (66.7%) | 9 (63.4%) |  | 52 (72.2%) | 17 (53.1%) | |  |
| **TMB** |  |  |  |  |  | |  |
| High | 18 (20.0%) | 1 (7.1%) | 0.643 | 16 (22.2%) | 3 (9.4%) | | 0.502 |
| low | 30 (33.3%) | 4 (28.6%) |  | 25 (34.7%) | 9 (28.1%) | |  |
| **Adjuvant therapy** | |  |  |  |  | |  |
| No | 7 (7.8%) | 1 (7.1%) | 1 | 5 (6.9%) | 3 (9.4%) | | 0.699 |
| Yes | 83 (92.2%) | 13 (92.9%) |  | 67 (93.1%) | 29 (90.6%) | |  |
| **Neoadjuvant therapy** | |  |  |  |  | |  |
| No | 77 (85.6%) | 13 (92.9%) | 0.687 | 61 (84.7%) | 29 (90.6%) | | 0.542 |
| Yes | 13 (14.4%) | 1 (7.1%) |  | 11 (15.3%) | 3 (9.4%) | |  |

**Table S2.** The comparison of gene variation frequency in cfDNA of recurrent and non-recurrent group from landmark MRD negative patients.

| **Gene** | **Not recurred** | | **Recurred** | | **Fisher’s exact test**  **(*P*)** |
| --- | --- | --- | --- | --- | --- |
|  | **Variation number** | **Frequency** | **Variation number** | **Frequency** |  |
| KRAS | 10 | 12.5% | 4 | 40.0% | **0.045** |
| EGFR | 8 | 10.0% | 2 | 20.0% | 0.307 |
| TP53 | 6 | 7.5% | 1 | 10.0% | 0.575 |
| FGFR3 | 7 | 8.8% | 2 | 20.0% | 0.261 |
| APC | 3 | 3.8% | 2 | 20.0% | 0.093 |
| ALK | 7 | 8.8% | 4 | 40.0% | **0.018** |
| FGFR2 | 6 | 7.5% | 2 | 20.0% | 0.216 |
| ERBB2 | 5 | 6.3% | 2 | 20.0% | 0.173 |
| ROS1 | 6 | 7.5% | 1 | 10.0% | 0.575 |
| RET | 4 | 5.0% | 3 | 30.0% | **0.028** |
| NTRK1 | 5 | 6.3% | 1 | 10.0% | 0.517 |
| GNAS | 6 | 7.5% | 0 | 0.0% | 1.000 |
| PIK3CA | 4 | 5.0% | 0 | 0.0% | 1.000 |
| DNMT3A | 4 | 5.0% | 1 | 10.0% | 0.453 |
| MET | 4 | 5.0% | 1 | 10.0% | 0.453 |
| NTRK3 | 2 | 2.5% | 2 | 20.0% | 0.059 |
| PDGFRA | 3 | 3.8% | 1 | 10.0% | 0.381 |
| EPAS1 | 2 | 2.5% | 1 | 10.0% | 0.301 |
| NOTCH1 | 3 | 3.8% | 0 | 0.0% | 1.000 |
| NTRK2 | 1 | 1.3% | 2 | 20.0% | **0.032** |
| MAPK3 | 2 | 2.5% | 0 | 0.0% | 1.000 |
| AR | 2 | 2.5% | 0 | 0.0% | 1.000 |
| ATM | 1 | 1.3% | 1 | 10.0% | 0.211 |
| KMT2D | 2 | 2.5% | 0 | 0.0% | 1.000 |
| BRD4 | 1 | 1.3% | 1 | 10.0% | 0.211 |
| KMT2B | 2 | 2.5% | 0 | 0.0% | 1.000 |
| PTPRS | 2 | 2.5% | 0 | 0.0% | 1.000 |
| RAD50 | 2 | 2.5% | 0 | 0.0% | 1.000 |
| FANCG | 1 | 1.3% | 1 | 10.0% | 0.211 |
| ERG | 0 | 0.0% | 1 | 10.0% | 0.111 |

**Table S3.** The comparison of gene variation frequency in cfDNA of recurrent and non-recurrent group from longitudinal MRD negative patients.

| **Gene** | **Not recurred** | | **Recurred** | | **Fisher’s exact test**  **(*P*)** |
| --- | --- | --- | --- | --- | --- |
|  | **Variation number** | **Frequency** | **Variation number** | **Frequency** |  |
| KRAS | 4 | 5.9% | 1 | 25.0% | 0.255 |
| EGFR | 7 | 10.3% | 1 | 25.0% | 0.382 |
| TP53 | 4 | 5.9% | 0 | 0.0% | 1.000 |
| FGFR3 | 1 | 1.5% | 1 | 25.0% | 0.109 |
| APC | 3 | 4.4% | 0 | 0.0% | 1.000 |
| ALK | 5 | 7.4% | 1 | 25.0% | 0.299 |
| FGFR2 | 4 | 5.9% | 0 | 0.0% | 1.000 |
| ERBB2 | 3 | 4.4% | 0 | 0.0% | 1.000 |
| ROS1 | 4 | 5.9% | 1 | 25.0% | 0.255 |
| RET | 3 | 4.4% | 1 | 25.0% | 0.208 |
| NTRK1 | 4 | 5.9% | 1 | 25.0% | 0.255 |
| GNAS | 5 | 7.4% | 0 | 0.0% | 1.000 |
| PIK3CA | 1 | 1.5% | 0 | 0.0% | 1.000 |
| DNMT3A | 2 | 2.9% | 0 | 0.0% | 1.000 |
| MET | 2 | 2.9% | 1 | 25.0% | 0.160 |
| NTRK3 | 1 | 1.5% | 0 | 0.0% | 1.000 |
| PDGFRA | 2 | 2.9% | 1 | 25.0% | 0.160 |
| EPAS1 | 1 | 1.5% | 1 | 25.0% | 0.109 |
| NOTCH1 | 3 | 4.4% | 0 | 0.0% | 1.000 |
| NTRK2 | 1 | 1.5% | 1 | 25.0% | 0.109 |
| MAPK3 | 2 | 2.9% | 0 | 0.0% | 1.000 |
| AR | 2 | 2.9% | 0 | 0.0% | 1.000 |
| ATM | 1 | 1.5% | 1 | 25.0% | 0.109 |
| KMT2D | 2 | 2.9% | 0 | 0.0% | 1.000 |
| BRD4 | 1 | 1.5% | 0 | 0.0% | 1.000 |
| KMT2B | 2 | 2.9% | 0 | 0.0% | 1.000 |
| PTPRS | 2 | 2.9% | 0 | 0.0% | 1.000 |
| RAD50 | 2 | 2.9% | 0 | 0.0% | 1.000 |
| FANCG | 1 | 1.5% | 0 | 0.0% | 1.000 |
| ERG | 0 | 0.0% | 1 | 25.0% | 0.056 |

1. Li, M.M., M. Datto, E.J. Duncavage, S. Kulkarni, N.I. Lindeman, S. Roy, et al. Standards and Guidelines for the Interpretation and Reporting of Sequence Variants in Cancer: A Joint Consensus Recommendation of the Association for Molecular Pathology, American Society of Clinical Oncology, and College of American Pathologists*.* *J Mol Diagn*. 2017;**19**:4-23. <https://doi.org/10.1016/j.jmoldx.2016.10.002>
